# A Gluten-Free Diet, Not an Appropriate Choice without a Medical Diagnosis

**DOI:** 10.1155/2019/2438934

**Published:** 2019-07-01

**Authors:** Ana Diez-Sampedro, Maria Olenick, Tatayana Maltseva, Monica Flowers

**Affiliations:** Undergraduate Nursing, NWCNHS, Florida International University, Miami, FL 33199, USA

## Abstract

In the past, only people diagnosed with celiac disease, approximately 1% of the population, avoided gluten consumption through all their meals. However, popular media often now mistakenly present gluten-free foods as being a healthier choice, and more people have now concluded that gluten is a harmful part of the diet. A review of literature on gluten-free diets, gluten sensitivity, celiac disease, and attitudes toward gluten consumption was undertaken to examine the prevalence and consequences of adopting a gluten-free diet and to provide guidance to healthcare practitioners whose patients are now often adopting this diet without medical input. Aside from celiac disease, nonceliac gluten sensitivity (NCGS) occurs in those persons in which gluten ingestion leads to symptomatic manifestations in the absence of celiac disease or wheat allergy but who report a remission of certain symptoms after removing gluten from their diet. However, it was been shown that a large percentage of people who claim NCGS do not feel those manifestations under a double-blind challenge to gluten. Moreover, some parents, believing that ingesting gluten is detrimental for their health, adopt gluten-free diets for their children. A review of existing data shows that there are detrimental effects to going gluten free, including loss of the dietary fiber, deficiencies in dietary minerals and vitamins, and potential heavy metal exposure. Healthcare practitioners should query patients about their dietary choices, and in cases of questionable adoption of gluten-free diet, patients and parents are educated about the detriments of a gluten-free diet, and in cases where patients continue to insist on gluten-free foods, referrals to nutritional counseling are warranted in order to minimize potential harm.

## 1. Increased Popularity of Gluten-Free Diets

There is an increasing body of evidence that a significant proportion of individuals, including not only persons with celiac disease (CD) or nonceliac gluten sensitivity (NCGS), are following a gluten-free diet (GFD). “Gluten free” was defined by the US Food and Drug Administration in 2013 as foods containing less than 20 parts per million of gluten or 20 mg/kg [1]. In fact, studies find that up to 5% of the population of Western societies report following a GFD of their own volition, while up to 13% self-report some sensitivity to gluten-containing products [2, 3]. With a GFD presented in popular media as a healthy diet, many people who do not have any symptoms after ingesting gluten appear to be adopting this diet. In the last few years, the popularity of a GFD has risen steadily in spite of stable rates of CD. Surprisingly, according to a survey by the NPD Group that was reported on National Public Radio, almost one-third of adult Americans say they would prefer to reduce or avoid gluten consumption altogether [4], compared to an estimated CD prevalence of only 1%. Similarly, a longitudinal study found that up to 13% of young adults valued gluten-free food, and these individuals were four to seven times more likely to value food production practices that were organic, local, non-GMO, and not processed [5]. In short, many individuals mistakenly believe that a GFD is an inherently healthier choice.

## 2. What Is Gluten?

Gluten is a high-molecular-weight seed storage protein commonly found in grass-related grains, such as wheat, barley, and rye. The natural role of these seed storage proteins is to nourish seeds during flowering and germination, thereby contributing to the successful reproduction of the species [6]. Gluten is a composite protein, composed of glutenin and prolamins, and is also responsible for the ability of wheat to form dough [7]. Hence, gluten is an integral component of an incredible variety of wheat-containing foods, including breads, cereals, and pastas.

## 3. Gluten-Related Disorders: Celiac Disease versus Nonceliac Gluten Sensitivity

Celiac disease (CD) affects approximately 1% of the population [8–10] and is an immune-mediated enteropathy most commonly associated with the human serotype HLA DQ2 or DQ8, with demonstration of small intestinal villous atrophy and autoantibodies (antitissue transglutaminase and antiendomysal) [11]. When people with CD ingest gluten, an inflammatory process that targets the intestinal mucosa is triggered, resulting in immune-mediated enteropathy with symptoms that may classically include malabsorption, diarrhea, steatorrhea, weight loss, or growth failure [11]. Studies have also suggested that some CD patients may not suffer from the classical gastrointestinal symptoms, but alternatively present with iron deficiency anemia [12], osteoporosis [13], ataxia or peripheral neuropathy [14], or symptoms that resemble IBD [15].

Nonceliac gluten sensitivity (NCGS) occurs in those persons in which gluten ingestion leads to symptomatic manifestations in the absence of celiac disease or wheat allergy (WA) but who report a remission of certain symptoms after removing gluten from their diet [16, 17]. Symptoms are related to the gastrointestinal track (abdominal pain, bloating, constipation, and diarrhea) or extraintestinal (fatigue, headache, joint and muscle pains, depression, and “foggy mind”) [18, 19]. Despite the name, it is not clear in many cases of NCGS whether the symptoms are caused by gluten itself or by other components of the grains that are consumed [20].

The Salerno Expert's Criteria provide guidance on how to test someone for NCGS. It is based on symptoms but follows a single- or double-blind placebo-controlled challenge [21]. A key element of these criteria is that the gluten and gluten-free preparations must be indistinguishable to the patient, which is difficult to achieve since gluten-free foods are typically detected easily due to differences in flavor and/or texture.

Lioneti et al. [22] found that the prevalence of NCGS after gluten rechallenge is decreased when the Salerno Expert's Criteria are applied. Their studies found that a high proportion of patients suspected to have a NCGS did not have symptoms in response to gluten ingestion when they were not able to distinguish between foods with or without gluten. They hypothesized that, in some patients, the symptoms suffered after gluten ingestion may be due to (among others) (a) psychological anticipation of intolerance (nocebo effect), (b) the possibility that NCGS may be a transient disorder, or (c) the methods used in the gluten challenge procedure.

## 4. Placebo and Nocebo Effects

Diagnosis of NCGS is very difficult, mostly because of the lack of validated diagnostic criteria and the high nocebo and placebo effects of gluten [23]. The true prevalence of NCGS is unknown, but several studies suggest that people may be diagnosed with NCGS when in reality they are not affected by gluten ingestion. Nocebo effect is often seen in patients that complain about intestinal or extraintestinal symptoms after ingesting gluten [21]. In fact, some studies have found that the placebo-nocebo effect is a key factor in those persons that report symptoms upon the ingestion of gluten and/or have been diagnosed with NCGS [24, 25]. Zanini et al. [25] studied subjects without CD or WA who fulfilled the clinical criteria of NCGS and adopted a GFD. They found that, after a double-blind gluten challenge, only one-third of patients still showed symptoms after ingesting gluten. It has been suggested that other proteins contained in wheat, such as lectins, agglutinin, or amylase trypsin inhibitors, may be responsible for triggering an innate immune response and leading to symptoms after wheat ingestion. Taken together, the data may indicate that, in many individuals who have not been conclusively diagnosed, the nocebo effect of gluten could be a reality, and they are following a GFD without a *bonafide* need to avoid gluten. It has even been suggested that it may be more appropriate to describe these patients as having “nonceliac wheat sensitivity” or “self-reported NCGS” in the absence of clinically confirmed gluten insensitivity [20, 26].

## 5. Physical Manifestations in People Suffering from NCGS

For those individuals that truly suffer from NCGS, the manifestations can be intestinal or extraintestinal. Intestinal manifestations include abdominal pain, bloating, and stool inconsistency, while extraintestinal manifestations can include fatigue, joint and muscle pain, and limb numbness. Moreover, neuronal manifestations can also present due to ingestion of gluten in some people. These manifestations include foggy mind, headache, gluten neuropathy, gluten encephalopathy, and gluten ataxia [27].

## 6. Detriments of a Gluten-Free Diet

### 6.1. High Cost and Inconvenience

Numerous studies have demonstrated that adherence to a GFD significantly increases the cost of food for adherents, with prices 2 to 3 times greater than for similar nongluten-free products [28–30]. Over 15.5 billion US dollars were spent on sales of gluten-free foods in 2016, double the amount spent in 2011, indicating that the popularity of a GFD continues to grow [31, 32]. In addition to cost, adherents of a GFD have inherently fewer choices in foods and may have difficulty finding or complying with a GFD [33]. Despite the significant cost and added inconvenience, healthy individuals that do not have CD or symptoms after ingesting gluten are increasingly willing to pay extra money for GF foods due to misplaced beliefs that those foods will make them healthier and feel better.

### 6.2. Nutritional Risks

GFD can lead to nutritional deficiencies of macronutrients and micronutrients. Gluten-free foods, when compared to equivalent wheat-based foods, show deficiencies in minerals, including calcium, iron, magnesium, and zinc, and in vitamins, including vitamin B12, folate, and vitamin D, as well as significantly reduced fiber [1, 34]. A 2013 study of both recently diagnosed and long-term celiac patients adhering to a GFD found nutritional deficiencies of each of these nutrients in both populations and recommended dietary education for celiac patients to help alleviate the problem [35]. In addition, as nonwheat substitutes in a GFD often contain little fiber, adherents of a GFD are at increased risk for constipation.

Notably, some food components known to be detrimental to health are higher in the GFD than in a regular diet. For example, a GFD increases dietary exposure to arsenic [36], and the meals tend to have a higher content of hydrogenated and saturated fatty acids and a higher glycemic index [17, 31, 34]. In general, it has been shown that a GFD is an unhealthier diet choice than a regular diet for those that do not have CD.

## 7. Benefits of a Gluten-Free Diet

For patients diagnosed with CD, a gluten-free diet is the only treatment. Ingestion of gluten in these genetically predisposed individuals results in a T-cell-mediated immune reaction, leading to villous atrophy and clinical symptoms. Avoiding gluten prevents this response, and as such, for CD patients, a gluten-free diet is critical to their well-being.

For sufferers of CD, the increase in popularity of a GFD among the general population has led to dramatically more selections of foods devoid of gluten, which are likely more palatable and more likely to be enriched with vitamins and minerals to meet the demands of an expanded marketplace. Also, more studies are aimed at finding those food sources that are still gluten free but higher in nutrients missing in a GFD.

For individuals who do not suffer from CD, benefits of a GFD are less obvious. The positive outcomes of a nocebo/placebo effect for those with NCGS, even if it is not the gluten component of wheat that leads to their symptoms, cannot be discounted. For some individuals, this may justify the detriments of a GFD.

The benefits of GFD continue to have an increased interest among researchers and practitioners working on the improvement of behavior in children with autism and other spectrum disorders. Even though some research informed that there was not significant evidence of improvement of functioning in children with autism, GFD was reported as well tolerated and safe [37]. Lee et al. [38] reported that modified gluten-free ketogenic diet with supplemental medium-chain triglycerides (MCT) has a positive treatment effect on the improvement of behavior in children with autism spectrum disorder and requires further investigation (p. 2).

## 8. NCGS in Children

As outlined above, there is a risk of nutritional deficiencies when either adults or children are subject to a GFD. As such, it is imperative to have the right diagnosis before placing any child on a GFD. For children with celiac disease, there is no other diet to follow than the GFD, yet diagnosis of CD requires that an individual continue to eat gluten prior to the diagnosis. After diagnosis, having a diet without gluten will help those children to heal their intestine and absorb more nutrients than if they are ingesting gluten.

In children that are diagnosed with NCGS, extra precaution needs to be taken because of the dangers of nutritional deficiency and the difficulty in achieving an accurate diagnosis absent a double-blind challenge, which is not typically achievable through a primary care healthcare provider. Tanpowpong et al. [39] found that the prevalence of gluten avoidance is 5 times higher than doctor-diagnosed celiac disease. Moreover, in a recent clinical trial in children with gastrointestinal and extra-intestinal symptoms related to gluten ingestion, it was found in a double-blind placebo-controlled (DBPC) gluten challenge that more than 60% of suspected cases were ruled out for NCGS diagnosis [19]. This confirms the need to acknowledge the presence of the nocebo effect when trying to diagnose NCGS, which could be as high as 40% [40], supporting the need to use a DBPC to establish the diagnosis. There is in fact limited data describing pediatric prevalence of NCGS, and its likelihood as a diagnosis is unclear [19].

Thus, the actual prevalence of NCGS does not appear to justify the commonality of a GFD in children, given the potential detriments of a GFD. In fact, there is currently no evidence to support the imposition of a GFD in asymptomatic children in the absence of CD or documented wheat allergy [41].

## 9. Implications for Nursing Practice

### 9.1. Assessment

It is necessary for pediatricians, nurse practitioners, and other health professionals working with children to query parents about their choice of diet for their children. As a sizable slice of the population has misidentified limiting gluten intake as being beneficial to their health, it seems to follow that those who subscribe to this belief and who become parents may feel similarly about what their children eat. It is common during routine exams for healthcare practitioners to ask about whether kids eat vegetables and fruits to get a sense of their diet. It is also important to establish whether kids eat grains to determine whether parents might be unnecessarily limiting their children to a GFD.

Although the long-term health effects of a GFD have not been established, a systematic review of prior studies has identified increased total cholesterol, high density lipoprotein, fasting glycemia, and body mass index as consistent findings [36]. Similarly, a recent population-based study found higher levels of heavy metal accumulation in adherents of a GFD, including higher blood levels of mercury, cadmium, and lead, as well as increased urine levels of arsenic [42]. Patients who do follow a GFD may therefore need assessment for clinical manifestations of a variety of nutritional deficiencies, including calcium, iron, magnesium, zinc, vit. B12, vit. D, and folate, as outlined above, as well as heavy metal toxicity, blood lipids, and glycemic levels.

### 9.2. Teaching

For parents of children with CD, it is a common practice for gastroenterologists and/or nutritionists to consult with parents to teach them the limitations of a GFD and to ensure that their children not only avoid gluten but also to teach parents how to secure the nutrients that are likely to be missing from their child's diet. Similarly, with the risk of a GFD in the general population, healthcare practitioners need to proactively question whether their patients, whether children or adults, are following a gluten-free diet of their own volition.

If patients, either adults or children, are suffering from symptoms that may be associated with CD, then referral to a gastroenterologist for proper diagnosis is critical. However, in the absence of symptoms, or vague descriptions of “gluten allergy” or other unrecognized disorders, patients who follow a GFD first need education on the true prevalence of CD and NCGS. They need to be taught of the need to secure sources of nutrients that may be limited in a GFD, and they need to know that following a GFD blindly may be harming themselves or their children. In the end, only persons with CD, WA, or confirmed NCGS should follow a GFD, and they should do so under medical supervision.

### 9.3. Referrals

Adults who self-administer a GFD and cite physical symptoms should be referred for CD testing. Adults who do not have CD but continue to insist on a GFD should be referred for nutritional counseling to ensure they are meeting their dietary needs and avoiding unnecessary hazards. Multivitamin/mineral and fiber supplements are a good idea for anyone attempting to avoid gluten to ensure adequate nutrition and to ensure proper bowel function.

For children who are put on a GFD, often by well-meaning parents, in the absence of a clinically relevant diagnosis, it is important that the parent be educated on the risks of a GFD, the need for CD testing if the parents claim symptoms in the child, and the potential hazards to their child of a GFD. If they insist, a referral to nutritional counseling is warranted.

## 10. Conclusions

While celiac disease patients must permanently remove gluten from their diet, the literature suggests that many individuals who claim to suffer from NCGS may in fact have no actual sensitivity to gluten although it is possible they are sensitive to other components of wheat. Alternatively, these individuals may be benefitting from a nocebo/placebo effect when it comes to adopting a GFD. It is likely, however, that the majority of persons adopting a GFD have no medical basis for doing so.

The choice to adopt a GFD is often made by individuals without medical input, in the belief that they are adopting a healthier diet. Unfortunately, choosing GFD, irrespective of whether it is medically indicated, generally entails increased food costs, a decrease in fiber consumption, potential decreases in mineral and vitamin consumption, including calcium, magnesium, zinc, vitamin B12, folate, and vitamin D, and potentially increased exposure to dietary hydrogenated and saturated fatty acids, and arsenic. Clearly, adopting a GFD does not come without risk.

In the end, only persons with CD, WA, or confirmed NCGS should follow a GFD, and they should do so under medical supervision. For individuals who insist on a GFD in the absence of a diagnosis that suggests its benefits outweighs its risks, nutritional counseling is recommended to ensure that these individuals can minimize the risks to themselves while still choosing the diet they prefer.
